# Interconnectedness between periodontitis stage, oral hygiene habits, adherence to the Mediterranean diet and nutritional status in Dalmatian kidney transplant recipients: a cross-sectional study

**DOI:** 10.1038/s41598-022-15589-6

**Published:** 2022-07-08

**Authors:** Josipa Radić, Marijana Vučković, Andrea Gelemanović, Marija Roguljić, Josip Orešković, Katja Kovačević, Ela Kolak, Dora Bučan Nenadić, Mislav Radić

**Affiliations:** 1 Department of Nephrology and Dialysis, University Hospital of Split, Spinčićeva 1, 21 000 Split, Croatia; 2 Department of Internal Medicine, University of Split School of Medicine, Šoltanska 2, 21 000 Split, Croatia; 3 Mediterranean Institute for Life Sciences (MedILS), Split, Croatia; 4 Department of Oral Medicine and Periodontology, School of Medicine, Study of Dental Medicine, University of Split, Šoltanska 1, 21000 Split, Croatia; 5 Private dental practice, 34000 Požega, Croatia; 6 Private dental practice, 21000 Split, Croatia; 7 Department of Nutrition and Dietetics, University Hospital Centre Split, Split, Croatia; 8 Division of Clinical Immunology and Rheumatology, Department of Internal Medicine, University Hospital of Split, Split, Croatia

**Keywords:** Health care, Medical research, Nephrology

## Abstract

The aim of this cross-sectional study was to determine the associations between the Mediterranean diet (MeDi), nutritional status parameters, muscle strength, and periodontal status in Dalmatian kidney transplant recipients (KTRs). 89 KTRs were included in this analysis, 40 (45%) women, with a mean age of 61 years (IQR = 13) and a mean time since kidney transplantation of 5 years (IQR = 6.6). An OHIP-14 questionnaire and questionnaire-based periodontal history were obtained from all participants, a comprehensive periodontal examination was performed. Body composition data, anthropometric and clinical parameters were collected for each study participant. The Mediterranean Diet Serving Score (MDSS) was used to assess MeDi adherence, and handgrip strength was measured with a hand dynamometer. Our results showed low adherence to MeDi in KTRs (28%) and almost 50% of KTRs suffer from severe forms of periodontitis. We also found a low OHIP-14 score and poor oral hygiene habits. KTRs with a less severe form of periodontitis had higher muscle mass and handgrip strength. MDSS score was associated with a higher number of teeth, and everyday cereal intake was inversely associated with the periodontitis stage. Our results demonstrate the associations between nutritional status, muscle strength, dietary habits, and periodontal health in Dalmatian KTRs.

## Introduction

Kidney transplantation (KTX) is considered the best treatment option for end-stage renal disease (ESRD)^1,2^. Despite the overall risk reduction, kidney transplant recipients (KTRs) are still at higher risk for infections as a side effect of immunosuppressive therapy^3^. In addition, the nutritional discrepancy between malnutrition due to long-term dialysis treatment and obesity due to numerous changes in the post-transplant period is also characteristic of this patient group^4,5^. In addition to classic risk factors and comorbidities such as post-transplant diabetes mellitus^6^ cardiovascular (CV) risk, infections, and malignancies, KTRs are at high risk of developing various oral diseases, with periodontitis (PD) being one of the most common^7^.

PD is one of the most common chronic oral diseases in adult population that affects supportive tooth tissues, causing their reduction and finally can lead to the tooth loss^8,9^. The effects of PD go beyond the oral cavity and the hematogenous spread of bacteria, their metabolic products, and mediators of inflammation from the periodontium affect distant tissues and organs^9^. Severe forms of PD are associated with the systemic chronic diseases such as CV diseases, diabetes, and chronic kidney disease (CKD) responsible for major number of deaths worldwide^9^. The significant body of evidence support the association between severe forms of PD and CKD indicating that people with PD have higher prevalence of CKD^9^ Systematic review of Nunes Dos Santos et al. indicated that periodontal status was associated with worsening graft function and systemic health among KTRs^10^. Considering immunosuppressive therapy and comorbidities of this specific patient population, it is not clear whether PD might present an additional risk to CKD in KTRs. Furthermore, nutritional status and poor dietary habits are in co-dependent relationship with oral health and disease, and they are associated with development of PD especially in elderly population^11,12^. Nutritional deficiencies in vitamins C and D and E, fatty acids, beta carotene, fiber, calcium, dairy, fruits and vegetables are associated with alerted tissue homeostasis and increased risk of PD^11,12^.

Previous research has found a bidirectional relationship between PD and CKD^13^, with inflammation, bacteremia, oxidative stress, protein-energy wasting, and hyperphosphatemia being some of the contributing factors^14^. Although there are reports of better oral health after KTX than ESRD, KTRs are at risk of developing and worsening PD due to the immunosuppressive therapy required^14^. Some studies suggest that periodontal status affects the survival of KTRs and graft survival^15–17^. Poor oral health has been associated with sarcopenia through the nutritional pathway of inadequate nutrient intake^18–20^, and in the other direction, sarcopenia could lead to a weakening of the oral musculature causing sarcopenic dysphagia^21,22^ thus completing a vicious cycle leading to the deterioration of overall health^23^.

In addition to oral health, nutritional status and dietary habits are often overlooked in this patient population. Recent studies have reported traditional Mediterranean dietary habits as protective for preserving renal graft function^24^, lower risk for development of comorbidities^25^, and a positive impact on nutritional status^26^. In the general population, the Mediterranean diet (MeDi) is reported to be associated with a lower risk of developing the periodontal disease^27,28^ and is considered an anti-inflammatory diet^29^. Eight-week adherence to the MeDi resulted in a decrease in PD associated pathogens in overweight and obese individuals^30^. To our knowledge, there are no comparable data for the KTR population. In our previous study, we found that the number of KTRs adhering to MeDi was alarmingly low^26^.

Since KTRs are at high risk of developing PD and PD is associated with greater health risks in this population, the aim of this study was to determine the associations between nutritional status, adherence to the MeDi and PD, and oral health in Dalmatian KTRs.

The primary hypothesis of our study was that KTRs with more severe forms of PD would have lower adherence to MeDi and its components. The secondary hypothesis was that KTRs with more severe forms of PD would have poorer nutritional status in terms of lower muscle mass and higher fat mass.

## Results

### Basic characteristics of study participants

**Figure 1:**
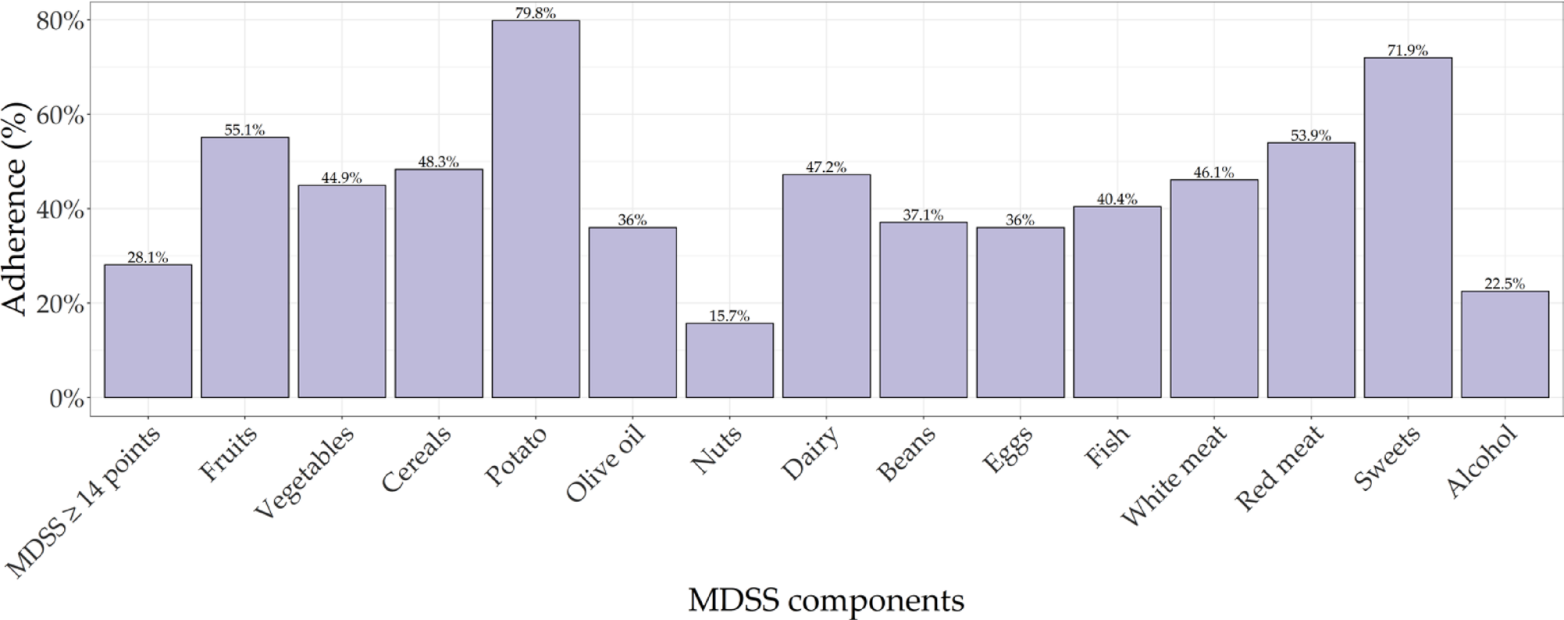
Mediterranean diet adherence in Dalmatian KTRs. *MDSS* Mediterranean Diet Serving Score.

Out of 101 KTRs with dental examination and data on MeDi adherence and body composition measurements, 89 (88%) of them had PD, 4 (4%) showed signs of gingivitis, while 8 (8%) were periodontally healthy. Due to the low numbers, we excluded participants without PD, thus, 89 KTRs were included in the final analysis. Out of those, there were 40 (45%) women and 49 (55%) men, with a median age of 61 years (IQR = 13). These KTRs had a median time since KTX of 5 years (IQR = 6.6), and their median eGFR, calculated using CKD-EPI, mL/min/1.73m^2^ was 45.2 (IQR = 25.6), 78 (88%) of them had arterial hypertension, 17 (19%) had diabetes mellitus, and 43 (48%) are ex or current smokers. Basic characteristics of study participants regarding anthropometric and body composition are shown in Table 1. Basic characteristics regarding adherence to the MeDi and individual components are shown in Fig. 1. Basic characteristics of study participants regarding periodontal status and oral hygiene habits are shown in Table 2.

**Table 1:** Basic characteristics of study participants regarding anthropometric and body composition parameters.

Variable	All KTRs (N = 89)
BMI (kg/m^2^), mean (SD)	26.62 (4.06)
Weight (kg), mean (SD)	80.24 (15.42)
MUAC (cm), median (IQR)	30 (7)
Waist circumference (cm), mean (SD)	100.58 (12.76)
WHtR, mean (SD)	0.58 (0.07)
Fat mass (kg), mean (SD)	19.72 (8.43)
Fat mass (%), mean (SD)	24.07 (8.52)
Fat free mass (kg), mean (SD)	60.78 (11.91)
Visceral fat, mean (SD)	9.63 (3.8)
Metabolic age, mean (SD)	50.88 (11.77)
Muscle mass (kg), median (IQR)	57.6 (18.2)
Skeletal muscle mass (kg), median (IQR)	32 (12.2)
Skeletal muscle mass (%), mean (SD)	41.1 (6.25)
Bone mass (kg), mean (SD)	3.03 (0.56)
Body water (kg), median (IQR)	41.7 (13.6)
Body water (%), mean (SD)	53.56 (6.3)
Phase angle, median (IQR)	5.1 (0.9)
ECW, mean (SD)	18.34 (3.01)
ICW, median (IQR)	23.9 (9.3)
ECW/ICW, median (IQR)	0.75 (0.13)
Trunk visceral fat, mean (SD)	10.38 (5.07)
Handgrip strength, median (IQR)	42 (19.05)

*BMI* body mass index, *MUAC* middle upper arm circumference, *WHtR* waist-to-height ratio, *ECW* extra cellular water, *ICW* intra cellular water.

**Table 2:** Basic characteristics of study participants regarding periodontal status and oral hygiene habits.

Variable	All KTRs (N = 89)
Dental visit (less than once a year), N (%)	36 (40.45)
Dental visit (once a year), N (%)	24 (26.97)
Dental visit (more than once a year), N (%)	29 (32.58)
Periodontist visit (no), N (%)	84 (94.38)
Periodontist visit (yes), N (%)	5 (5.62)
Daily teeth brushing (less than once a day), N (%)	26 (29.21)
Daily teeth brushing (once a day), N (%)	48 (53.93)
Daily teeth brushing (more than once a day), N (%)	15 (16.85)
Toothbrush and toothpaste (no), N (%)	5 (5.62)
Toothbrush and toothpaste (yes), N (%)	84 (94.38)
Dental floss (no), N (%)	75 (91.46)
Dental floss (yes), N (%)	7 (8.54)
Interdental brushes (no), N (%)	80 (97.56)
Interdental brushes (yes), N (%)	2 (2.44)
Toothpicks (no), N (%)	67 (81.71)
Toothpicks (yes), N (%)	15 (18.29)
Mouth wash (no), N (%)	77 (86.52)
Mouth wash (yes), N (%)	12 (13.48)
Bleeding while brushing (no), N (%)	47 (59.49)
Bleeding while brushing (yes), N (%)	32 (40.51)
Bad breath (no), N (%)	59 (66.29)
Bad breath (yes), N (%)	30 (33.71)
Loose tooth (no), N (%)	59 (74.68)
Loose tooth (yes), N (%)	20 (25.32)
Dental plaque removal (less than once a year), N (%)	55 (67.9)
Dental plaque removal (once a year), N (%)	19 (23.46)
Dental plaque removal (more than once a year), N (%)	7 (8.64)
Loss of tooth (other reasons), N (%)	50 (56.18)
Loss of tooth (due to periodontitis), N (%)	39 (43.82)
Number of teeth, median (IQR)	14 (15)
Dental plaque (%), median (IQR)	90 (38.5)
Bleeding (%), median (IQR)	11 (27.5)
Average pocket depth, median (IQR)	1.99 (0.84)
Average total CAL, median (IQR)	2.85 (1.33)
Average interdental CAL, median (IQR)	2.90 (1.33)
Periodontitis stage (mild), N (%)	46 (51.69)
Periodontitis stage (severe), N (%)	43 (48.31)
OHRQoL, median (IQR)	7 (10)

*CAL* clinical attachment loss, *OHRQoL* oral health related quality of life.

### Associations between dental and nutritional parameters in Dalmatian KTRs

**Figure 2:**
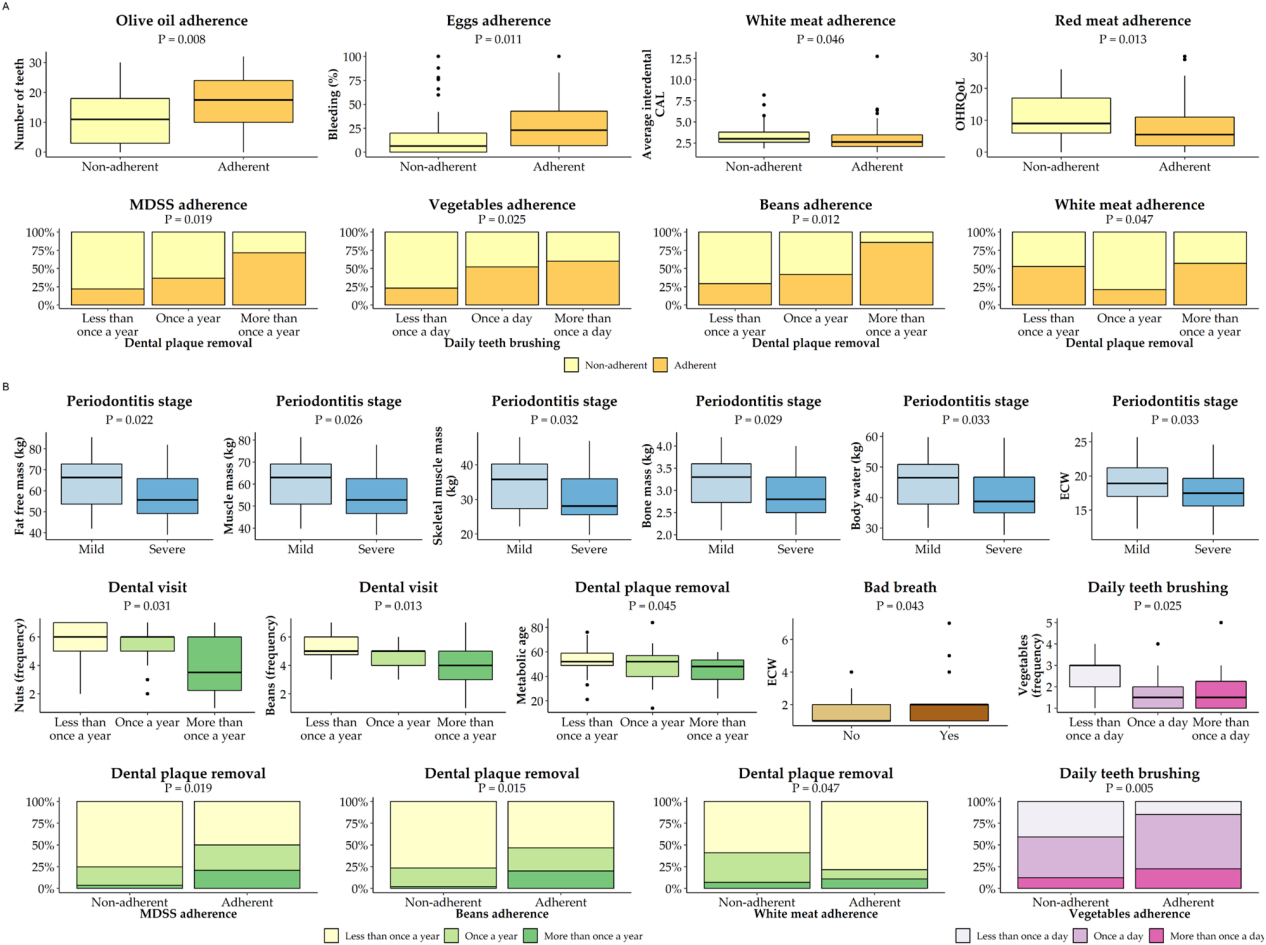
Significant associations between dental, MeDi and body composition parameters based on descriptive statistics. (**A**) Categories based on adherence to MeDi and its components; (**B**) categories based on categorical dental parameters. *ECW* extracellular water, *MDSS* Mediterranean Diet Serving Score, *CAL* clinical attachment loss; *OHRQoL* Oral Health Related Quality of Life. Color coding according to the categories of specific parameters: orange—adherence vs non-adherence to MeDi and its components; blue—mild vs severe periodontitis stage; green—yearly dental visit or dental plaque removal; purple—daily teeth brushing; brown—presence vs no bad breath. MeDi components depicted as frequency ranges from 1 to 7; 1—two or more times a day, 7—rarely or never.

As the main aim of this study was to assess if there are any associations between the dental and nutritional parameters (nutritional parameters being adherence to MeDi and its componence and various anthropometric and body composition parameters), the ideal setting would be to compare KTRs with PD with KTRs without PD. However, due to the very high prevalence of PD in this group of patients, it was impossible to gather enough KTRs without PD, thus this study was designed as a cross-sectional study. To examine the possible associations between dental and nutritional parameters, we performed multiple case-control designs as subset analyses where we grouped KTRs based on either nutritional or dental categorical parameters and in this way the same group of KTR patients served as their own control. We first explored the descriptive statistics after grouping the participants according to the different groups of nutritional and dental categorical parameters. Nutritional categories were BMI (< 25.0, 25–29.9, ≥ 30.0) and adherence recommendations to MeDi and its components (14 in total: fruits, vegetable, cereals, potato, olive oil, nuts, dairy, beans, eggs, fish, white meat, red meat, sweets, alcohol), while dental categories were PD stage (mild, severe), PD localization (localized, generalized), the reason for tooth loss (PD, other), dental visit and removing dental plaque (less than once a year, once a year, more than once a year), daily teeth brushing (less than once a day, once a day, more than once a day), presence of bleeding while brushing, presence of bad breath and presence of loose tooth. Statistically significant results are summarized in Fig. 2, with (A) showing the groups based on adherence to MeDi and its components, and (B) showing the groups based on categorical periodontal variables. Full results are given in Supplementary Table S1. There were several significant associations between the periodontal and nutritional parameters, all in the direction of better adherence to MeDi and its components and better periodontal status. Some of the key findings are that KTRs more adherent to MeDi and beans recommendations are removing their dental plaque more frequently and those adherent to vegetable recommendations are brushing their teeth once or more than once a day, while those adherent to olive oil recommendations are having statistically significantly more teeth. When we looked at periodontal categories, some of the key findings were that KTRs with severe PD have less fat-free mass, less muscle mass, less skeletal muscle mass, less body mass, less body water and lower ECW. KTRs that go to regular yearly dental check-ups are also more adherent to the recommendations to eating nuts and beans, while those that do regular dental plaque removal are also adherent to the overall MeDi and beans recommendations. Finally, participants adhering to recommendations for eating vegetables are also cleaning their teeth more regularly.

**Figure 3:**
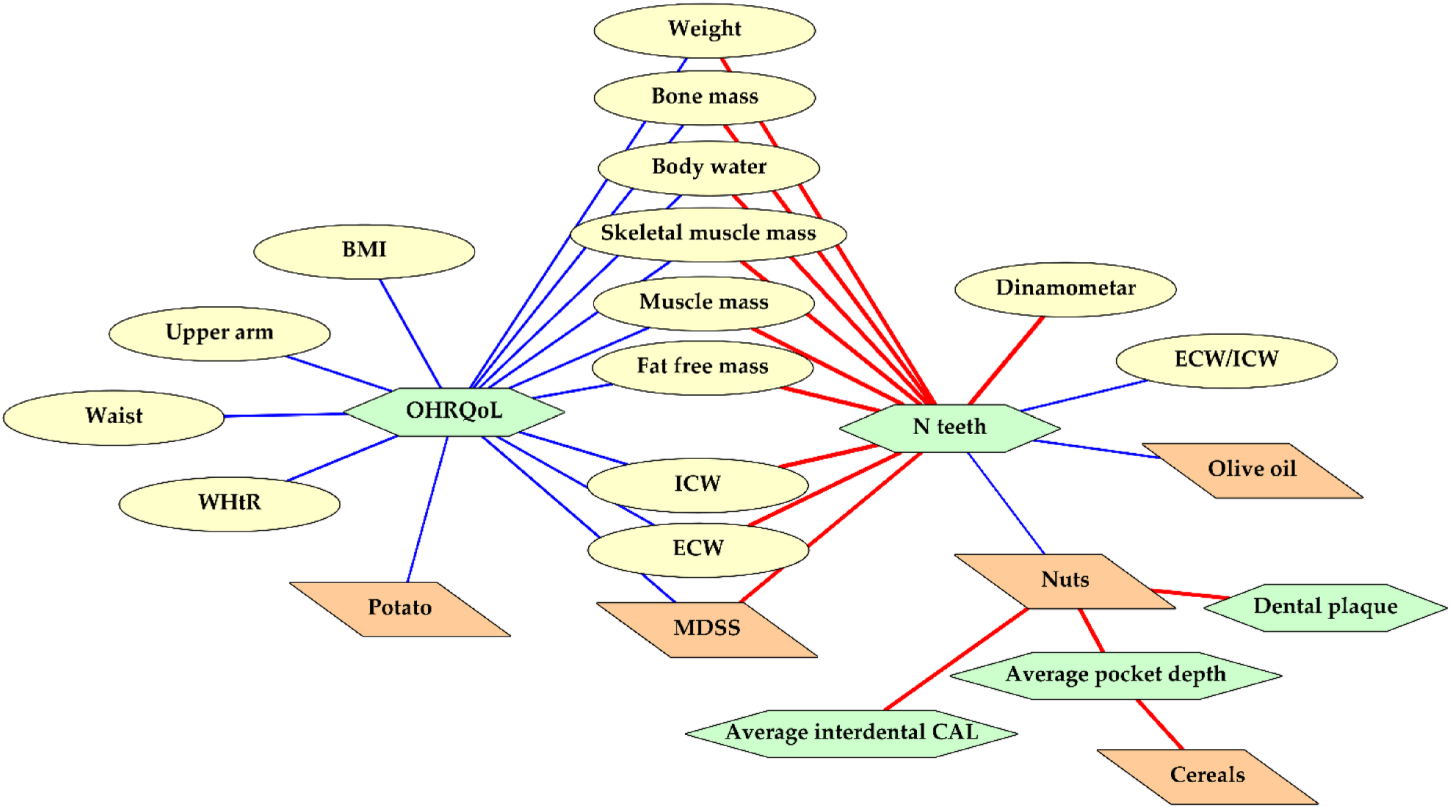
Significant associations between dental, MeDi and body composition parameters based on Spearman’s rank correlation. Abbreviations: *BMI* body mass index, *WHtR* waist-to-height ratio, *ECW* extracellular water, *ICW* intracellular water, *MDSS* Mediterranean Diet Serving Score, *CAL* clinical attachment loss; *OHRQoL* Oral Health Related Quality of Life. Color coding: yellow ellipses are body composition parameters; orange parallelograms are overall MeDi score and frequency of eating each MeDi component (frequency ranges from 1 to 7; 1—two or more times a day, 7—rarely or never); green hexagons are dental parameters; red lines represent significant positive correlation; blue lines represent the significant negative correlation.

Association between numerical parameters regarding nutritional and dental status was additionally examined with Spearman’s rank correlation analysis and statistically significant results are depicted in Fig. 3, while the full table of results is shown in Supplementary Table S2. A positive correlation was observed between oral health related quality of life (OHRQol) and various anthropometric and body composition parameters, while a negative correlation was observed with a number of teeth, meaning that worse periodontal status, in general, is associated with poorer nutritional status. When we examined the frequency of consumption of individual MeDi components, KTRs that consume olive oil and nuts rarely or never have statistically a smaller number of teeth. Frequent consumption of nuts was associated with lower dental plaque, interdental CAL and pocket depth, together with frequent consumption of cereals.

To examine which nutritional factors are associated with the severe PD stage, a multivariate logistic regression analysis was performed, and the results are shown in Table 3. Additionally, we performed multivariate linear regression with a number of teeth as the dependent variable. Both regression models were adjusted for potential confounders which are age, sex, transplantation years, eGFR, presence of arterial hypertension, presence of diabetes mellitus, smoking status, yearly dental visit, and daily teeth brushing. Adherence to cereals recommendations were the only statistically significant protective factor for severe PD stage (OR = 0.35, 95% CI 0.13–0.94, P = 0.037). If we examine periodontal status based on the number of teeth, better periodontal status (meaning more teeth) was associated with better handgrip strength (beta = 0.109, SE = 0.045, P = 0.018), better adherence to the overall MeDi score (beta = 0.460, SE = 0.195, P = 0.021) and adherence to olive oil recommendations (beta = 5.843, SE = 1.579, P < 0.001).

**Table 3:** Multivariate linear and logistic regression of parameters associated with higher number of teeth and severe periodontitis stage.

Predictor	Number of teeth			Periodontitis stage		
	Beta	SE	P*	OR	95% CI	P**
Weight	0.088	0.063	0.167	0.99	0.96–1.03	0.677
BMI (kg/m^2^)	0.280	0.204	0.174	0.99	0.89–1.11	0.874
MUAC (cm)	− 0.149	0.138	0.285	1.04	0.96–1.12	0.341
Waist circumference (cm)	0.083	0.073	0.258	0.98	0.94–1.02	0.347
WHtR	16.766	12.817	0.195	0.05	0–70.56	0.427
Fat mass (kg)	0.114	0.102	0.270	0.98	0.93–1.04	0.564
Fat mass (%)	0.104	0.113	0.360	0.98	0.92–1.05	0.631
Fat free mass (kg)	0.078	0.117	0.508	1	0.94–1.06	0.940
Visceral fat	0.109	0.259	0.675	0.98	0.85–1.13	0.778
Metabolic age	0.079	0.096	0.411	0.99	0.94–1.05	0.749
Muscle mass (kg)	0.103	0.125	0.413	0.98	0.92–1.05	0.648
Skeletal muscle mass (kg)	0.086	0.178	0.631	1	0.91–1.1	0.971
Skeletal muscle mass (%)	− 0.059	0.149	0.692	1.01	0.93–1.1	0.783
Body mass (kg)	0.801	2.435	0.743	1.07	0.28–4.08	0.918
Body water (kg)	0.128	0.158	0.420	0.99	0.91–1.08	0.881
Body water (%)	− 0.128	0.150	0.397	1.02	0.94–1.11	0.693
Phase angle	0.872	0.881	0.325	1.11	0.7–1.77	0.660
ECW	0.152	0.392	0.699	0.98	0.79–1.21	0.835
ICW	0.187	0.214	0.385	1.01	0.9–1.14	0.826
ECW/ICW	− 3.016	14.243	0.833	0.06	0–165.79	0.491
Trunk visceral fat	0.155	0.167	0.356	0.97	0.88–1.06	0.490
Handgrip strength	0.109	0.045	**0.018**	0.99	0.96–1.01	0.269
MDSS	0.460	0.195	**0.021**	0.98	0.88–1.09	0.733
MDSS adherence	2.417	1.855	0.197	1.06	0.38–2.95	0.915
Fruits adherence	0.045	1.755	0.980	0.8	0.31–2.06	0.644
Vegetables adherence	1.812	1.849	0.330	1.16	0.42–3.19	0.774
Cereals adherence	3.239	1.653	0.054	0.35	0.13–0.94	**0.037**
Potato adherence	1.588	2.099	0.452	1.45	0.45–4.68	0.533
Olive oil adherence	5.843	1.579	** < 0.001**	0.76	0.3–1.95	0.571
Nuts adherence	1.274	2.325	0.585	1.36	0.38–4.79	0.636
Dairy adherence	− 2.335	1.682	0.169	2.04	0.8–5.23	0.137
Beans adherence	1.413	1.775	0.428	1.31	0.49–3.51	0.596
Eggs adherence	1.282	1.717	0.458	0.55	0.21–1.44	0.220
Fish adherence	− 0.298	1.694	0.861	1.5	0.59–3.8	0.394
White meat adherence	− 1.351	1.766	0.447	1.33	0.51–3.5	0.559
Red meat adherence	1.192	1.716	0.489	0.78	0.31–1.99	0.606
Sweets adherence	− 0.629	1.905	0.742	2.59	0.86–7.73	0.089
Alcohol adherence	2.322	2.106	0.274	0.87	0.28–2.77	0.819

*BMI* body mass index, *MUAC* middle upper arm circumference, *WHtR* waist-to-height ratio, *ECW* extra cellular water, *ICW* intra cellular water, *MDSS* Mediterranean Diet Serving Score, *SE* standard error, *OR* odds ratio, *CI* confidence interval.

*Multivariate linear regression model.

**Multivariate logistic regression model; both models adjusted for potential confounders age, sex, transplantation years, eGFR, presence of arterial hypertension, presence of diabetes mellitus, smoking status, yearly dental visit, and daily teeth brushing; statistically significant variables are depicted in bold.

## Discussion

To our knowledge, this is the first study to examine associations between MeDi, body composition parameters, and periodontal health in KTRs. Our previous results indicate low adherence to MeDi in Dalmatian KTRs^26^, which is also the case in this sample, where MeDi adherence is only 28.09%. Low adherence to MeDi is also reported in the healthy Dalmatian population. Pribisalic et al.^31^ reported MeDi adherence of 18.5% among 4671 adults from Dalmatia, and Dragun et al.^32^ reported MeDi adherence of only 12.1% among adolescents and medical students in Dalmatia. MeDi adherence is also low among residents of the Croatian islands. Kolčić et al.^33^ reported adherence of 18.8% on the island of Korčula and 31.9% on the island of Vis, questioning the existence of the MeDi in southern Croatia. The prevalence of PD in this group of patients was quite high (88%) and almost 50% of Dalmatian KTRs had a severe stage of PD, while only 5 (5.62%) of them had been previously examined by a periodontist. Unfortunately, KTR patients do not have a high interest in periodontal disease screening and/or treatment. Similarly to our findings, Schmalz et al^34^ showed that the majority of KTRs had clinically moderate (47%) and severe PD (32%). In addition, their results showed that periodontal treatment was necessary in 71% of KTRs.

In addition, our results suggest that 40.45% of KTRs visit their dentist less than once a year and almost 30% of them brush their teeth less than once a day. Regarding other dental care products, 91.46% of KTRs do not floss, 98% of them do not use interdental brushes, while the use of toothpicks and mouthwash occurs in a slightly higher number of KTRs, 18.29% and 13.48%, respectively. 40.51% of KTRs reported bleeding while brushing their teeth and 33.71% of participants had bad breath.

Interestingly, the median OHIP-14 score was 7 out of 56, which is rather low and indicates a high quality of life in terms of oral health in Dalmatian KTRs, despite reported dental health problems. The years on dialysis and the complex procedure of KTX might be the reason why dental health problems are not considered a priority in this population of patients. Like our results, Schmalz et al. also reported a low importance of oral health in KTRs^35^. All these results suggest that Dalmatian KTRs have low adherence to MeDi and poor oral hygiene habits. In addition, education about lifestyle changes is needed in the care of KTRs. Dental care of KTRs needs to be improved. OHIP-14 scores indicate that low importance is subjectively attached to oral health, resulting in the need to motivate and raise awareness in this patient group.

Regarding body composition parameters, our results showed significantly higher levels of fat free mass, muscle mass, and skeletal muscle mass in KTRs with less severe forms of PD. Consistent with this is the association between higher handgrip strength and number of teeth from our regression analyses.

Tooth loss affects diet quality and nutrient intake in ways that may increase the risk of developing sarcopenia. Similarly to our findings, Chang Hoon Han et al.^36^ have shown that sarcopenia is associated with tooth loss in the geriatric population because tooth loss often leads to decreased oral function, such as impaired eating and chewing ability^37^, which ultimately reduces muscle mass and quality of life^38^. These authors also suggested that the associations between tooth loss and low muscle mass may occur through inflammatory^39^ and nutritional pathways^40^. Therefore, they showed that the presence of PD was significantly higher in participants with sarcopenia and the number of natural teeth was significantly lower in participants with sarcopenia. The explanation for this finding may be that inflammatory cytokines, common in PD, activate many of the molecular signaling pathways involved in skeletal muscle wasting, leading to an imbalance between protein synthesis and protein catabolism^36^.

Our results showed an association between MDSS score and a higher number of teeth in KTRs. It can be assumed that the MeDi has a positive effect on the oral health of KTRs, but conversely, we could assume that KTRs with more teeth and better oral health are more likely to eat hard foods such as raw fruits, vegetables, and nuts, as suggested by the MeDi. We found no significant associations between the MDSS score and other PD parameters, whereas the study by Laiola et al. adherence to the MeDi over an eight-week period resulted in a decrease in PD-associated pathogens in overweight and obese individuals^30^. Other studies in the general population also found an association between MeDi adherence and periodontal health^27,41^. In a recent study examining the association between certain known dietary patterns and the prevalence of periodontal disease in a northern population-based cohort study, results showed that participants with low adherence to the MeDi had a significantly higher plaque index and more cases of severe PD overall than participants with high adherence to the diet^42^.

The results of our regression analyses also showed an association between adherence to daily olive oil consumption according to MeDi and a higher number of teeth. In a study of 1075 young Moroccan subjects by Iwasaki et al.^43^ no significant association was found between MDSS and PD. However, consumption of olive oil, a component of MeDi, showed a significant inverse association with PD.

Daily consumption of cereals showed an inverse association with PD stage in Dalmatian KTRs. These results are consistent with studies on US Hispanics that found an inverse association between whole-grain consumption and PD^44^.

Our results suggest that KTRs who adhere more to MeDi and bean recommendations remove their plaque more frequently and those who adhere to vegetable recommendations brush their teeth once or more than once a day, while those who adhere to olive oil recommendations have statistically significantly more teeth. In addition, KTRs who attend regular dental checkups are also more likely to adhere to recommendations for nut and bean consumption. All these findings suggest an association between healthy eating habits and healthy oral health habits among KTRs.

In our study population, we found poor plaque control, but we did not find gingival hyperplasia among the participants, although all of them underwent continuous immunosuppressive therapy, including the calcineurin inhibitor cyclosporine. The scope of our study was not to investigate the effects of immunosuppressive therapy on the severity of PD. However, it would be appropriate to conduct such studies in the future, as it is known from the literature that cyclosporine-induced gingival overgrowth increases plaque accumulation, which is the most important factor in PD^45,46^.

This study has some general limitations, mainly resulting from the cross-sectional design, which as such does not allow for causal relationships. In addition, this study lacks a control group of KTRs without PD. Because of the very high prevalence of PD in KTRs, it was not possible for our center to enroll enough KTRs without PD to form the proper control group for this study. Another limitation is that this is a single-center study, but with a representative study population due to the large number of people coming to our center. Another limitation of this study is that we did not consider data on other factors that might influence body composition, such as exercise and daily caloric and protein intake. In this study, oral health assessment was limited to periodontal tissue examination. Considering that dietary habits have an impact on caries formation^47^, future studies should be conducted to investigate the effects of MeDi on hard dental tissues as well.

The results of our study indicate a relationship between oral health, nutritional status, and MeDi adherence in Dalmatian KTRs. Low MeDi adherence and poor oral hygiene habits lead us to conclude that more attention should be paid to educating KTRs about the importance of oral health and nutrition. The role of a healthy lifestyle in maintaining good nutritional and dental health is particularly important for the KTR population.

To the best of our knowledge, we found no data in the literature on the prevalence of PD in KTRs. Like our study design, in the study by Hyeon-Jin Min et al, which examined the effect of PD on posttransplant outcomes in KTRs, all participants were diagnosed as PD cases and were divided into two groups according to the severity of disease^48^. Part of the explanation for the high prevalence of PD in KTRs may be that this patient population had several risk factors for PD, such as older age, poor oral hygiene, and several comorbidities. However, further studies are needed to investigate the prevalence of periodontal disease in this specific patient population. Further prospective multicenter studies with larger numbers of participants are needed to determine the causal relationships more accurately between oral health, nutritional status, and diet in the KTRs population.

## Materials and methods

The detailed protocol of our study and the general measurements were described in our previously published paper^26^.

In addition to the measurements described in our previous study, we also conducted a detailed examination of dental health. Participants in this study were KTRs with functioning grafts who were willing to undergo a dental examination. In addition to dental status, we were also interested in MeDi adherence and body composition in this study^26^**.** The study was conducted according to the guidelines of the Declaration of Helsinki, and approved by Ethics Committee of University Hospital of Split on 30 August 2019. (Ur.no. 2181-147-01/06/M.S.-19-2, Class: 500-03/19-01/72.). Informed consent was obtained from all subjects involved in the study.

### Body composition and anthropometric measurement

Body composition was assessed using an MC-780 Multi Frequency Segmental Body Analyzer (Tanita, Tokyo, Japan) for each study participant. The device uses a constant high-frequency current flow and eight electrodes to determine the electrical resistance of different tissues. The method is called bioelectrical impedance analysis (BIA). It is used to assess fat mass (kg), fat mass percentage (%), fat-free mass (kg), visceral fat, muscle mass (kg), skeletal muscle mass (kg), skeletal muscle mass percentage (%) and body mass (kg). All patients were advised not to take any food or liquid at least 3 h before the measurement, to urinate just before the measurement and not to consume alcohol, eat or drink excessively nor to exercise in an excessive way at least one day before the body composition measurement^49^. Height was measured using a stadiometer. Waist circumference (WC) and mid-upper arm circumference (MUAC) were measured using a flexible, non-stretchable measuring tape in a standing position facing forward with their shoulders relaxed. Body mass index (BMI) and the waist-to-height ratio (WHtR) were calculated for each study participant.

Handgrip strength was measured three times using a hand dynamometer (Saehan, Korea) and an average value was calculated.

### Mediterranean diet serving score

The Validated Mediterranean Diet Serving Score (MDSS) questionnaire was used to determine adherence to the Mediterranean Diet (MeDi) considering the consumption of different foods and food groups (MeDi components) in time intervals per meal, day or week^50^.

The food is divided into fourteen food groups, and points are given according to the new Mediterranean food pyramid in the following way: three points for fruits, vegetables, olive oil and cereals if consumed with each meal; Two points for dairy products and nuts if consumed daily; One point for the recommended number of servings per week is consumed for potatoes (≤ 3), legumes (≥ 2), eggs (2–4), fish (≥ 2), white meat (2), red meat (< 2), sweets (≤ 2) and fermented beverages (1–2 glasses a day)^17^.

It sums up to a maximum MDSS score of twenty-four (24), and the greater score implies greater adherence to the MeDi. The optimal cut-off point ≥13.50 was set to determine adherence or non-adherence to the MeDi^50^.

### Medical history, clinical and laboratory parameters

By thorough examination of patients medical records, data about the existence and duration of primary CKD, arterial hypertension, diabetes mellitus, cardiovascular events as well as the time of kidney transplantation (KTX), type and duration of dialysis treatment before KTX were obtained.

Regarding laboratory parameters, all study participants underwent usual peripheral blood sampling, and they were asked to obtain a 24-h urine sample on the same day as the body composition and blood pressure measurement. We collected data on levels of urea (mmol/L), creatinine (mmol/L), uric acid (mmol/L), serum albumin (g/L), phosphates (mmol/L), C-reactive protein (CRP; mg/L), calcium (mmol/L), glucose (mmol/L), triglycerides (mmol/L), total cholesterol (mmol/L), low-density lipoprotein cholesterol (LDL) (mmol/L), haemoglobin (g/L), mean cellular volume (MCV), sodium (mmol/L), potassium (mmol/L), and eGFR using CKD-EPI (mL/min/1.73 m2). A complete blood count was obtained using a hematology analyser (Advia 120, Siemens, Erlangen, Germany) and intact parathyroid hormone (PTH; pmol/L) was measured by immunoassay analyzer (Cobas e601, Roche Diagnostics, Penzberg, Germany).

### Periodontal health assessment

Questionnaire**-**based periodontal anamnesis was taken from all participants. Participants were asked about the frequency of dental and periodontist visits, dental plaque removal, frequency of washing their teeth and the use of oral hygiene products (e.g. usage of toothbrush, toothpick, dental floss, interdental brushes**)**. They were also asked about the presence of symptoms such as bad breath, bleeding while brushing and loose teeth. A comprehensive periodontal examination was performed by an experienced periodontist (MR) using the UNC 15 mm periodontal probe (Aesculap, Tuttlingen, Germany). All clinical parameters measured on six sites were recorded: the full mouth plaque score (FMPS), bleeding on probing (BOP), probing pocket depth (PPD), gingival recession (GR) and clinical attachment level (CAL). Periodontal variables were considered periodontal stages according to the new classification scheme as it was proposed in the paper of Tonetti et al.^51^. Mild PD included stages I and II, while severe was classified as stages III and IV. If the participant reported a loss of > 4 teeth due to PD or had no teeth it was grouped within the severe PD group.

### Oral health related quality of life (OHrQoL) assessment

The self-administered Croatian version of the OHIP**-**14 questionnaire was used to measure the dental outcome in terms of its influence on OHrQoL^52^**.** The OHIP-14 questionnaire is a 14-item measure with statements divided into 7 theoretical domains: functional limitation, pain, psychological discomfort, physical disability, psychological disability, social disability, and handicap^53^. Responses to each of the 14 statements are as follows**:** 0 = never, 1 = hardly ever, 2 = occasionally, 3 = often, and 4 = very often. The maximum score is 56 and lower scores indicate better**,** while higher scores indicate worse OHrQoL.

### Statistical analyses

Categorical variables were reported as numbers with percentages and different groups were compared using the chi-square test. Numerical data were first tested for normality using the Shapiro-Wilk test, and if the variables were normally distributed it was reported as means with standard deviation (SD) and compared using either t-test or Anova as appropriate, while if the variables were not distributed normally, it was reported as medians with interquartile range (IQR) and compared using Mann-Whitney U or Kruskal-Wallis test as appropriate. Spearman’s rank correlation analysis was performed to examine the association between numerical variables. Finally, multivariate logistic and linear regression analyses were performed with PD stage and number of teeth as dependent variables, respectively. Independent variables were anthropometric and body composition parameters, as well as adherence to overall MeDi and its individual components. Both regression models were adjusted for possible confounders: age, sex, years since KTX, estimated glomerular filtration rate (eGFR, calculated using CKD-EPI, mL/min/1.73m^2^), presence of arterial hypertension and diabetes mellitus, smoking status, yearly dental visit, and daily teeth brushing. Results of linear regression were shown as betas with standard errors (SE), while results of logistic regression were shown as odds ratios (OR) with a 95% confidence interval (CI). The significance level was set at 0.05 for all analyses. All analyses were performed using the free software environment for statistical computing, R version 4.0.0^54^. Network of significant correlations between numerical variables was designed using Cytoscape software v3.7.1.^55^.

## Supplementary Information

Supplementary Information.

## Data Availability

Raw data is available at corresponding author mail: mislavradic@gmail.com.
